# Supplementary material for: Whole-Genome Resequencing of Ujimqin Sheep Identifies Genes Associated with Vertebral Number
Source: Animals (Basel). 2024 Feb 21;14(5):677. doi: 10.3390/ani14050677 (PMC10931107; doi:10.3390/ani14050677)
Supplement: Supplementary file 1 [file animals-14-00677-s001.zip › Figure S1.pdf]

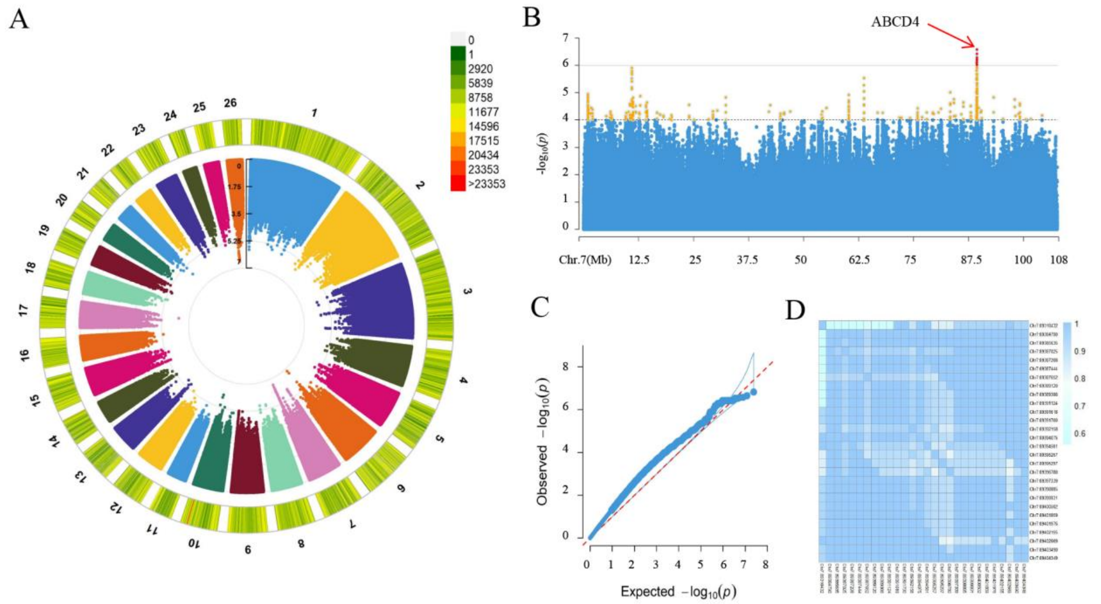

Genome-wide association study for vertebrae number in Ujimqin sheep. (**A**) Annular Manhattan plots of GWAS results and SNP density distribution map on 1–26 chromosomes. The X axis shows SNPs across chromosomes SSC1 to SSC26, and the Y axis represents the −log10(p). (**B**) Manhattan plots of SSC7 of GWAS results. The top line indicates the genome-wide significant thresholds ($p < 10^{-6}$). (**C**) QQ plot of GWAS analysis. (**D**) Heat map of linkage disequilibrium between Significant SNP on SSC7. The X axis and Y axis is SNPs.
